# Age, growth and population structure of invasive lionfish (*Pterois volitans/miles*) in northeast Florida using a length-based, age-structured population model

**DOI:** 10.7717/peerj.2730

**Published:** 2016-12-01

**Authors:** Eric G. Johnson, Mary Katherine Swenarton

**Affiliations:** Department of Biology, University of North Florida, Jacksonville, FL, United States

**Keywords:** Lionfish, Invasive species, Growth, Length-based modeling, *Pterois volitans*

## Abstract

The effective management of invasive species requires detailed understanding of the invader’s life history. This information is essential for modeling population growth and predicting rates of expansion, quantifying ecological impacts and assessing the efficacy of removal and control strategies. Indo-Pacific lionfish (*Pterois volitans/miles*) have rapidly invaded the western Atlantic, Gulf of Mexico and Caribbean Sea with documented negative impacts on native ecosystems. To better understand the life history of this species, we developed and validated a length-based, age-structured model to investigate age, growth and population structure in northeast Florida. The main findings of this study were: (1) lionfish exhibited rapid growth with seasonal variation in growth rates; (2) distinct cohorts were clearly identifiable in the length-frequency data, suggesting that lionfish are recruiting during a relatively short period in summer; and (3) the majority of lionfish were less than two years old with no lionfish older than three years of age, which may be the result of culling efforts as well as ontogenetic habitat shifts to deeper water.

## Introduction

Invasive species are organisms that have been introduced to areas where they do not naturally occur, and whose establishment adversely affect native biotas and ecosystems. The establishment of an invasive species can have far reaching effects on invaded ecosystems through predation (Race, 1982), competition for prey or habitat (Mills et al., 1993; Byers, 2009), or by introducing novel pathogens or parasites (Crowl et al., 2008). Ultimately, invasives can lead to declines in the abundance and diversity or even extinction of native organisms (Grosholz et al., 2000) with cascading effects on ecosystem structure and function (Vitousek, 1990). Historically, research on biological invasions has focused heavily on terrestrial plant species (Lowry et al., 2013), yet invasions in marine ecosystems have received increasing attention (Verling et al., 2005; Rilov & Crooks, 2009). Recent interest in marine systems has been driven in part by high profile marine invasions (e.g., *Carcinus maenus* (Carlton & Cohen, 2003), *Caleurpa taxifola* (Meinesz, 1999), *Didemnum vexillum* (Lambert, 2009)) and because the principal vectors of marine introductions (e.g., commercial shipping, aquaculture, aquarium trade) have increased dramatically in magnitude (Minchin et al., 2009).

A marine invasive species of particular concern is the lionfish (*Pterois volitans/miles*), now established throughout the western Atlantic, Gulf of Mexico, and Caribbean Sea (Schofield, 2009; Morris Jr, 2012; Dahl & Patterson, 2014). Lionfish have long venomous spines that deter predation, exhibit rapid growth (Barbour et al., 2011; Edwards, Frazer & Jacoby, 2014; Pusack et al., 2016), mature early and reproduce year-round (Morris Jr, 2009), and are capable of long distance dispersal during egg and larval stages (Ahrenholz & Morris, 2010; Johnston & Purkis, 2011). This combination of life history characteristics has allowed this species to establish and spread rapidly (see Côté, Green & Hixon, 2013 and references therein for review); lionfish are now among the most abundant predatory fishes in many areas of the invaded range (Whitfield et al., 2007; Dahl & Patterson, 2014). Lionfish prey on an array of reef fishes (Morris Jr & Akins, 2009) and are capable of reducing native fish recruitment by nearly 80% (Albins & Hixon, 2008) and overall fish biomass by 65% (Green et al., 2012) with potential cascading impacts on ecosystem structure and function, including extirpations (Albins, 2015; Ingeman, 2016). Current evidence suggests that biotic resistance is not likely to present a significant barrier to the lionfish invasion (Albins, 2013; Hackerott et al., 2013; Valdivia et al., 2014) prompting human intervention to help control this species. Harvest is now actively promoted by management agencies throughout the western Atlantic Ocean and Caribbean Sea and has shown promise in reducing densities of this invasive species at local scales (Frazer et al., 2012; Albins & Hixon, 2013; de León et al., 2013; Green et al., 2014).

Previous empirical and modeling studies suggest high levels of sustained removal effort will be required to reduce lionfish biomass and minimize impacts (Barbour et al., 2011; Morris Jr, Shertzer & Rice, 2011; Johnston & Purkis, 2015). Many of these studies employ population modeling approaches that rely on key estimates of life history (e.g., growth, mortality, fecundity) with considerable uncertainty that may also vary in space or over time (Fogg et al., 2015). Moreover, these models, which typically evaluate the effects of varying control and harvest strategies on lionfish population density or biomass (Barbour et al., 2011; Johnston & Purkis, 2015) and the response of native fish communities (Green et al., 2014), can be highly sensitive to changes in life history inputs (Morris Jr, Shertzer & Rice, 2011). Consequently, considerable effort has been directed at estimating vital rates for lionfish in the invaded range. In particular, growth has been the focus of numerous studies (Potts, Berrane & Morris Jr, 2010; Barbour et al., 2011; Jud & Layman, 2012; Benkwitt, 2013; Akins, Morris Jr & Green, 2014; Edwards, Frazer & Jacoby, 2014; Fogg et al., 2015; Rodríguez-Cortés, Aguilar-Perera & Bonilla-Gómez, 2015; Pusack et al., 2016). Growth is critically important since body size strongly influences predator–prey interactions (Rice et al., 1993; Sogard, 1997; Lorenzen, 2006), winter mortality in temperate species (Henderson, Holmes & Bamber, 1988; Hurst, 2007) and is a key determinant of reproductive output in fishes (Hixon, Johnson & Sogard, 2013).

Current estimates of lionfish growth are available from the temperate Atlantic (Potts, Berrane & Morris Jr, 2010; Barbour et al., 2011), Gulf of Mexico (Fogg et al., 2015; Rodríguez-Cortés, Aguilar-Perera & Bonilla-Gómez, 2015) and Caribbean Sea (Edwards, Frazer & Jacoby, 2014; Pusack et al., 2016), but are unavailable in many regions including the southern South Atlantic Bight which was one of the first areas to be colonized after introduction (Schofield, 2009). Because growth can vary with ecological factors (e.g., population density, food availability) and the abiotic environment (e.g., temperature), spatially-explicit estimates are often required to describe growth accurately. Previous age and growth estimates for lionfish have typically been generated from analyses of otoliths (Potts, Berrane & Morris Jr, 2010; Barbour et al., 2011; Edwards, Frazer & Jacoby, 2014; Fogg et al., 2015) or tagging (Jud & Layman, 2012; Akins, Morris Jr & Green, 2014; Pusack et al., 2016); both being effective but time consuming and effort intensive methodologies. Estimating growth from length-frequency data has a long history in fisheries management and can be an effective alternative approach, particularly when age data are sparse or not available (Pauly & David, 1981; Pauly & Morgan, 1987; Fournier et al., 1990); and has recently been applied to lionfish (Rodríguez-Cortés, Aguilar-Perera & Bonilla-Gómez, 2015). Herein, we describe a flexible length-based, age-structured model to provide insight into the life history of lionfish in northeast Florida. The purpose of this study was to (1) construct a statistical length-based model for estimating age, growth and population structure of lionfish, (2) evaluate the performance of our model using validation from otolith analysis and external length-frequency data, and (3) provide estimates of vital rates and population structure of lionfish in northeast Florida where such information is not currently available.

## Methods

### Ethics statement

All lionfish used in this study were handled in strict accordance with a UNF IACUC protocol (IACUC#13-004) and tissues of opportunity waivers approved by the University of North Florida. UNF IACUC defines tissues of opportunity as samples collected: (1) during the course of another project with an approved IACUC protocol from another institution; (2) during normal veterinary care by appropriately permitted facilities; or (3) from free-ranging animals by appropriately permitted facilities. Lionfish removals are encouraged by the State of Florida and sample collection locations did not require any specific permissions. No endangered or protected species were harmed during the course of this study.

### Sample collections

**Figure 1:**
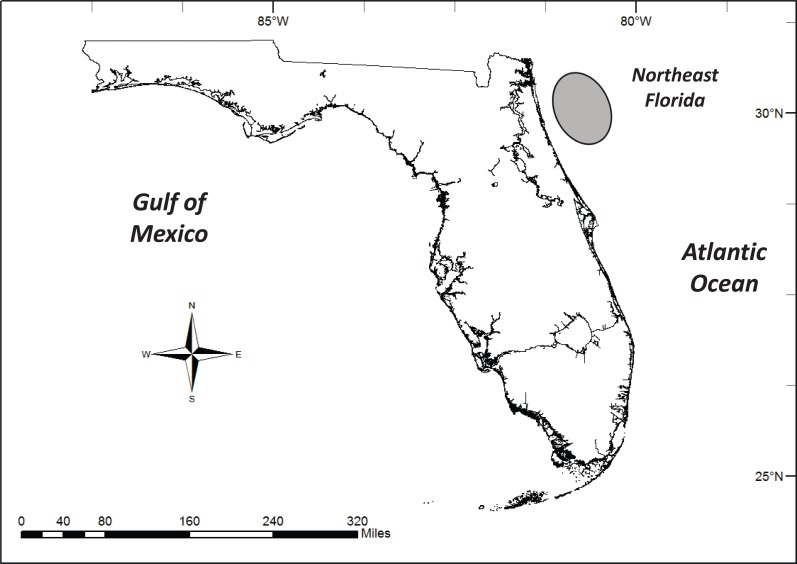
Lionfish collection region (shaded oval) off the coast of northeast Florida.

Lionfish were collected from locations offshore of northeast Florida by trained spearfishermen (Fig. 1). Sampling occurred primarily during several large-scale public removal events in 2013, 2014 and 2015 (April and August) and during opportunistic sampling by recreational spearfishermen in 2014 (July, September, October, November) and early 2015 (January). All lionfish were captured offshore (>15 km) on hardbottom and artificial reef habitats in approximately 20–35 m of depth. Lionfish were measured in the field for standard (SL) and total length (TL) to the nearest 1 mm. A subset of lionfish from each tournament, and all lionfish from the opportunistic samples, were transported to the University of North Florida for further processing. In the laboratory, lionfish were measured (SL and TL), weighed to the nearest 0.1 g and sexed. Some small lionfish were difficult to accurately sex and were considered immature (Morris Jr, 2009). A subset of lionfish (*n* = 100) had their sagittal otoliths removed, which were used to determine lionfish age directly and in model validation.

### Length-based, age-structured model

**Figure 2:**
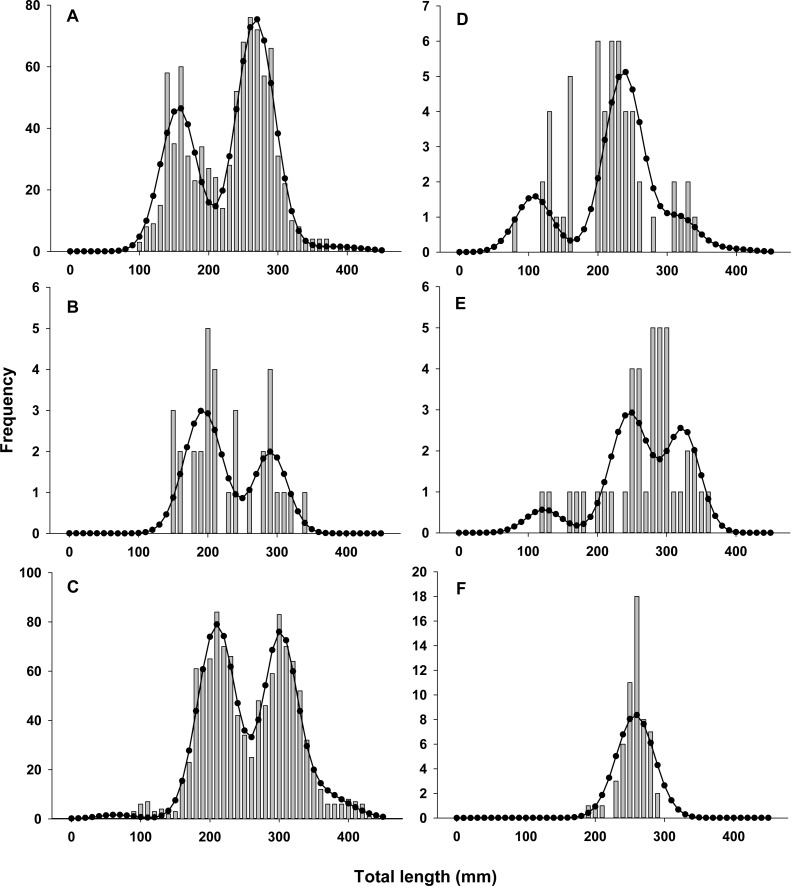
Observed length frequency histograms of lionfish collected from northeast Florida used in model parameterization. Length-frequency of lionfish collected in (A) April 2014, (B) July 2014, (C) August 2014, (D) October 2014, (E) November 2014, and (F) January 2015 (grey bars). The black curve in each panel symbolizes the predicted length frequency distribution of lionfish from the best fit candidate model (Model 1, see Table 1).

Lionfish TL data from 2014 were used to construct length-frequency histograms for the observed data from 0 to 450 mm (TL) using 10 mm increments (46 length bins) for each collection month separately (Fig. 2). Length-frequency data from 2013 and 2015 were not used in model fitting because of low temporal resolution; however these data were used to assess model performance (see *Model validation*). Because sex was not determined for many lionfish, length-frequency data were pooled. Growth and population age structure were estimated by fitting a length-based, age-structured model to the observed length-frequency data.

**Table 1:** Summary of model diagnostics and fit for four candidate population models. Model diagnostics (−ln (L), number of parameters (*K*), Akaike Information Criteria (AIC), corrected AIC (AIC_*c*_), and model weights) for the four candidate models. Seasonal indicates whether a seasonalized (Yes) or traditional (No) von Bertalanffy growth function was fit to the data. Variance at age indicates whether variance in size-at-age was held constant across ages (Fixed) or allowed to vary among ages (Variable).

Model	Seasonal	*σ*^2^ at age	−ln(L)	*K*	AIC	AIC_*c*_	ΔAIC_*c*_	*ω*_*i*_
1	Yes	Variable	648.92	32	1361.84	1370.53	0.00	1.00
2	Yes	Fixed	659.09	29	1376.18	1383.26	12.72	0.00
3	No	Variable	667.38	30	1394.76	1402.35	31.82	0.00
4	No	Fixed	672.25	27	1398.51	1404.60	34.07	0.00

The model uses length as a proxy for age and estimates the proportion of fish of a given size in each age class using a maximum likelihood approach by fitting a predicted length frequency distribution to the observed data (Pauly & David, 1981; Johnson, 2004). To generate the predicted length-frequency distribution, the mean size-at-age was first estimated using either the traditional (von Bertalanffy, 1938; Beverton & Holt, 1957) or seasonalized (Somers, 1988; García-Berthou et al., 2012) formulation of the von Bertalanffy growth function (VBGF) which expresses fish length as a function of age. The traditional formulation of the VBGF is given below (Eq. 1):

(1) \begin{eqnarray*}{L}_{t}={L}_{\infty } \left[ 1-{e}^{- \left( K \left( t-{t}_{0} \right) \right) } \right] \end{eqnarray*}

where *L*_*t*_ is the length of a fish at age *t*, *L*_∞_ is the asymptotic maximum length, *K* is the Brody growth coefficient, and *t*_0_ is the theoretical time at which a fish was length 0. The seasonalized VBGF (Eq. 2) extends the traditional VBGF to allow the growth rate to vary seasonally, and may better reflect the growth of fishes in temperate regions which experience pronounced seasonal temperature fluctuations. The seasonalized VBGF (Somers, 1988) is given in the series of equations below (Eqs. 2–4):

(2) \begin{eqnarray*}{L}_{t}={L}_{\infty } \left[ 1-{e}^{- \left( K \left( t-{t}_{0} \right) -S \left( t \right) +S \left( {t}_{0} \right) \right) } \right] \end{eqnarray*}

(3) \begin{eqnarray*}S \left( t \right) = \left( \frac{CK}{2\pi } \right) \sin \nolimits \,\pi \left( t-{t}_{s} \right) \end{eqnarray*}

(4) \begin{eqnarray*}S \left( {t}_{0} \right) = \left( \frac{CK}{2\pi } \right) \sin \nolimits \,\pi \left( {t}_{0}-{t}_{s} \right) \end{eqnarray*}

where *L*_*t*_, *L*_∞_, *K*, *t*_0_ are the same as previously defined (Eq. 1), *t*_*s*_ sets the timing of seasonal growth oscillations relative to *t*_0_ (*t*_*s*_ + 0.5 = winter point (*t*_*w*_); the time of slowest growth), and *C* is the intensity of seasonal growth oscillation (0 ≤ *C* ≤ 1). A value of *C* = 0 indicates no seasonality in growth, while *C* = 1 indicates extreme seasonality and complete cessation of growth at *t*_*w*_. Because the VBGF only estimates the mean size-at-age over time; variation in the size of individual fish within each age class was estimated by including variance in size-at-age (${\sigma }_{a}^{2}$) as a model parameter. The proportion of lionfish in each age class during each sampling month and year was also estimated within the model (*P*_*a*,*t*_). The expected number of individuals of age *a* in size class *i* in month *t* (*n*_*a*,*i*,*t*_) was then calculated:

(5) \begin{eqnarray*}{n}_{a,i,t}={N}_{t}\cdot {P}_{a,t}\cdot P \left( {L}_{lower}\leq {L}_{i}\leq {L}_{upper}\hspace*{1em} \left\vert \right. N({\overline{L}}_{a,t},{\sigma }_{a}^{2}) \right) \end{eqnarray*}

where *N*_*t*_ is the total number of individuals captured in month *t*, *P*_*a*,*t*_ is the probability of a fish captured in month *t* being age *a*, *L*_*lower*_ and *L*_*upper*_ are the lower and upper bounds of a predicted 10 mm size bin (e.g., 230 and 240 mm), and $N({\overline{L}}_{a,t},{\sigma }_{a})$ defines a normal probability density function with the mean length ${\overline{L}}_{a,t}$ of fish of age *a* in month *t* estimated from the VBGF (Eqs. 1 or 2–4), and model estimated standard deviation at age, *σ*_*a*_. Because size distributions overlap across ages, the total number of expected fish of size *i* in month *t* was then calculated by summing the expected contributions to size class *i* from each age:

(6) \begin{eqnarray*}{N}_{i,t}=\sum _{a=0}^{3}{n}_{i,a,t}.\end{eqnarray*}

Four different candidate models were compared based on all combinations of two possible model structures (1) seasonal versus non-seasonal growth and (2) fixed versus variable variance in size-at-age (Table 2). Model fit was assessed by varying model parameters to minimize the log-likelihood between observed and predicted (Eq. 6) monthly length-frequency data using the SOLVER optimization routine in Microsoft Excel (MS Excel 2013, Microsoft, Inc. Seattle, WA, USA). Akaike’s Information Criterion corrected for sample size (AIC*c*) was used to select the best model from the candidate set and quantify the relative support of each model given the data (*ω*_*i*_).

**Table 2:** Parameter estimates from a length-based, age structured population model. (Top) Model parameter estimates (von Bertalanffy growth parameters, estimated date of birth and variation in size-at-age) for lionfish from northeast Florida estimated from four candidate models (see Table 1). (Bottom) Results of sensitivity runs using the best fit model (Model 1); parameter values were fixed at −10%/+10% of best fit estimates to examine the effect on remaining model parameters. As in the best fit model base run, *L*_∞_ was also fixed in most sensitivity runs to ensure biologically realistic parameter values.

Candidate models		VBGF parameters				DOB	*σ* (size at age)			
Seasonal	*σ*^2^ at age	*L*_∞_	*K*	*C*	*t*_*s*_	*t*_*b*_	Age 0	Age 1	Age 2	Age 3
Yes	Variable	448^*^	0.47	0.61	0.21	0.42	26.29	27.48	24.13	40.30
Yes	Fixed	448^*^	0.46	0.63	0.24	0.40	26.47	26.47	26.47	26.47
No	Variable	448^*^	0.47	n/a	n/a	0.36	28.41	27.24	25.21	37.21
No	Fixed	448^*^	0.46	n/a	n/a	0.34	26.87	26.87	26.87	26.87

Sensitivity runs		*L*_∞_	*K*	*C*	*t*_*s*_	*t*_*b*_	Age 0	Age 1	Age 2	Age 3
*L*_∞_ (−10%)		403.2^*^	0.59	0.54	0.14	0.49	26.45	27.27	24.21	29.29
*L*_∞_ (+10%)		492.8^*^	0.39	0.66	0.27	0.35	26.19	27.57	24.12	48.03
*K* (−10%)		472.6	0.42^*^	0.58	0.23	0.38	26.37	27.51	24.07	45.08
*K* (+10%)		429.5	0.52^*^	0.58	0.17	0.45	26.38	27.46	24.17	37.50
*C* (−10%)		448^*^	0.47	0.55^*^	0.20	0.42	26.30	27.48	24.13	40.33
*C* (+10%)		448^*^	0.47	0.67^*^	0.21	0.42	26.28	27.48	24.13	40.28
*t*_*s*_ (−10%)		448^*^	0.47	0.34	0.13^*^	0.40	26.30	27.52	24.12	40.75
*t*_*s*_ (+10%)		448^*^	0.46	0.68	0.28^*^	0.38	26.97	27.13	24.29	35.48
*t*_*b*_ (−10%)		448^*^	0.46	0.31	0.14	0.38^*^	26.42	27.41	24.08	38.33
*t*_*b*_ (+10%)		448^*^	0.47	1.00	0.20	0.46^*^	26.26	27.57	24.22	42.39

**Notes.**

* Parameter value was fixed to ensure biologically realistic values (see text for details).

The key assumptions of all candidate models were as follows: (1) predicted length-at-age follows a normal distribution with mean ${\overline{L}}_{a}\hspace*{1em}$ and standard deviation*σ*_*a*_, (2) there are only four age classes present in the observed length-frequency samples (age 0, 1, 2, and 3; this assumption was verified by aging of sagittal otoliths from a subset of lionfish (*n* = 100) which found that only 8% of individuals were age three (despite non-random sampling that was biased to select larger individuals), and no individuals were age four or older (see *Model Validation*), and (3) lionfish recruitment is assumed to occur at a single point during the year and was estimated in the model by the parameter, *t*_*b*_, the estimated birth date of an annual cohort. This simplifying assumption is surely violated to some degree (every fish is not born on the same day), yet model outputs are not significantly affected because *t*_*b*_ in essence represents the average birth date with the variance in size-at -age (${\sigma }_{a}^{2}$) reflecting a combination of variation in birth dates confounded with the variability in growth rates among individual fish. We further assumed that (4) because diver effort varied across time and the pattern of selectivity for lionfish of varying ages is unknown, the proportion of lionfish in each age class may not accurately reflect overall population structure. While this assumption does not directly impact our estimates of model parameters, it does limit the some of the conclusions that can be drawn from the data. As a result, no attempt is made to make quantitative inferences regarding relative changes in abundance of cohorts between years or over time (e.g., recruitment strength, natural mortality).

### Model performance and sensitivity

Two types of analyses were conducted to examine the robustness of our model and associated parameter estimates: (1) a randomized grid search designed to evaluate the ability of the model to converge on a consistent solution from randomly generated sets of initial parameter values (±25% best fit values) and (2) the sensitivity of model outputs was assessed by fixing individual model parameters at ±10% of their best fit values, allowing the model to converge on a new constrained solution, and examining the resulting effect on model fit and parameter estimates.

### Model validation

Direct aging of a 100 fish subsample using sagittal otolith analysis was performed to verify ages and provide external validation for model outputs. Otoliths were extracted by first making a transverse cut into the skull, and removing the otoliths from under the brain cavity. Otoliths were rinsed and stored dry in envelopes until processing. Aging analysis was conducted by the Florida Fish and Wildlife Research Institute (FWRI) following the procedures outlined in VanderKooy & Guindon-Tisdel (2003). Briefly, a singular otolith from each fish was embedded in casting resin and 500 µm sections were cut using a Buehler low-speed Isomet saw. Sections were then mounted on glass slides with histomount and viewed under reflected light with a dissecting microscope at 32× magnification. Marginal increment analysis was conducted and the distance from the most recent annulus to the otolith margin was scored 1–4. Two readers aged the otoliths independently. If the ages did not agree, the otolith was removed from further analysis (*n* = 7). Otolith ages were then reconciled with the model chronology using Eq. (7) which generates the actual age (*A*_*a*_) at the time of the first annulus formation in years (time elapsed from the estimated date of birth (*t*_*b*_) to deposition of the first annulus):

(7) \begin{eqnarray*}\hspace*{1em}{A}_{a}={t}_{w}+1-{t}_{b}\end{eqnarray*}

where *A*_*a*_ is the actual fractional age in years, *t*_*w*_ is the winter point from the seasonal VBGF, and *t*_*b*_ is the model estimated birth date. This adjustment is valid assuming that annuli deposition is coincident with the winter point (*t*_*w*_) when growth is slowest. To generate ages for older fish (1+) we simply added a year for each additional annulus present. To evaluate model performance, the VBGF from the best fit model was plotted against observed size-at-age from otolith analysis to examine the level of agreement in the two independent measures of growth.

**Figure 3:**
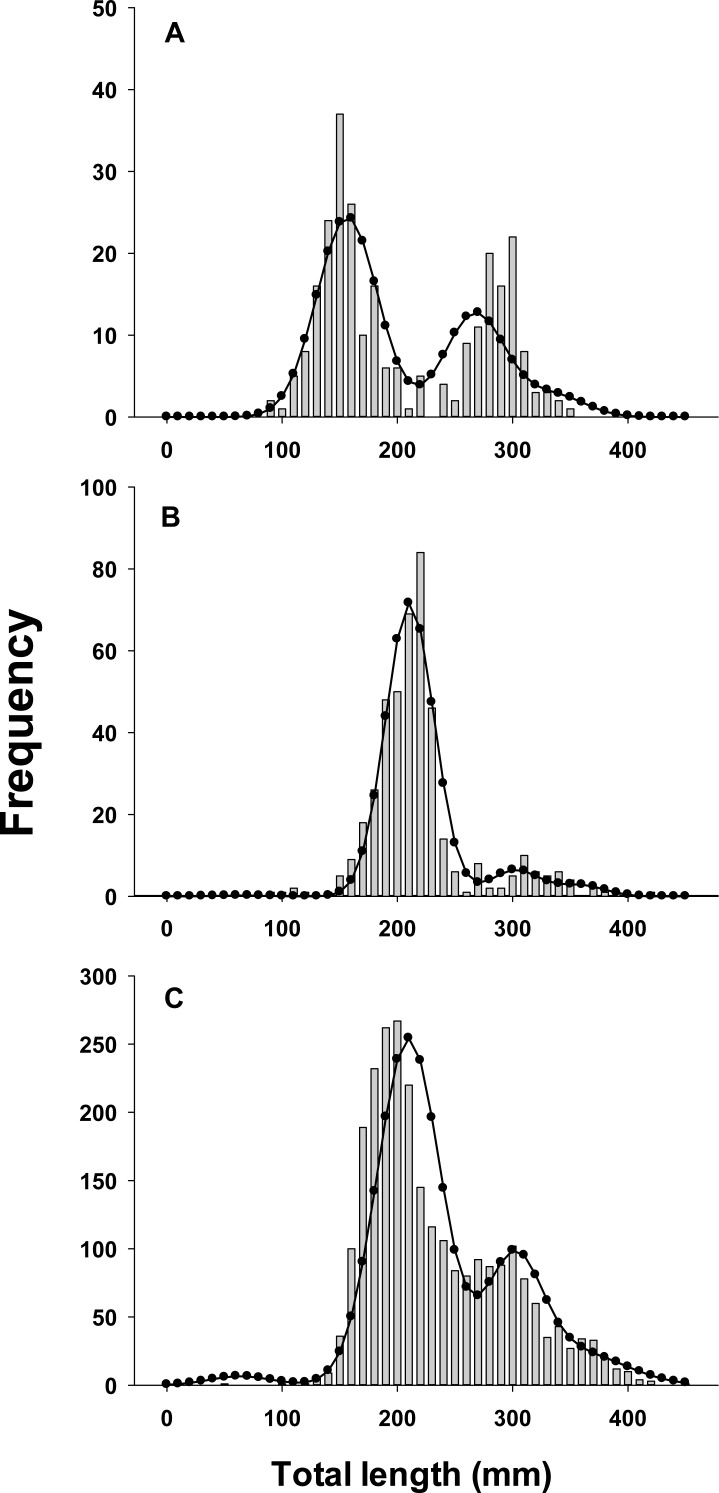
Observed length frequency histograms of lionfish collected from northeast Florida used in model validation. Length-frequency of lionfish collected in (A) April 2013, (B) April 2015, (C) August 2015 (grey bars). The black curve in each panel symbolizes the predicted length frequency distribution of lionfish from the best fit candidate model (Model 1, see Table 1). Observed data were not used in model fit.

As a further evaluation of model performance, we fit the best model to three sets of observed lionfish length-frequency data from northeast Florida that were not used in parameter estimation and thus external to the model (Fig. 3). These were not used in model fitting because available samples in those years (2013, 2015) lacked sufficient temporal resolution to evaluate seasonal growth models.

## Results

### General

The total lengths of lionfish sampled (*n* = 2, 137) ranged from 41 to 448 mm in northeast Florida over the study period. Maximum length (448 mm) and minimum length (41 mm) were both recorded in August of 2014. Some fish were not sexed and some fish were immature, but of the fish that were sexed there were 466 females present and 727 males.

### Model selection

There was almost complete support for model 1 as the best fit model (*ω*_*i*_ ≈ 1), which assumed seasonal variability in growth and age dependent variance in size-at-age (Table 1). Models that did not assume seasonal variability in growth or had fixed variance in size-at-age fit the data poorly and had essentially no support (*ω*_*i*_ ≈ 0). The best model fit observed length-frequency distributions well (Fig. 2), particularly in months with large sample sizes. The best model converged on biologically realistic values for life history parameters (Table 2) with the exception of *L*_∞_ (see below). A grid search using randomly generated initial parameter values consistently converged on the same set of parameter values indicating a global minimum and robust solution were reached. Sensitivity analyses revealed that parameter estimates were generally robust with the exception of *L*_∞_ and *k* which were inversely correlated (Table 2).

### Growth

**Figure 4:**
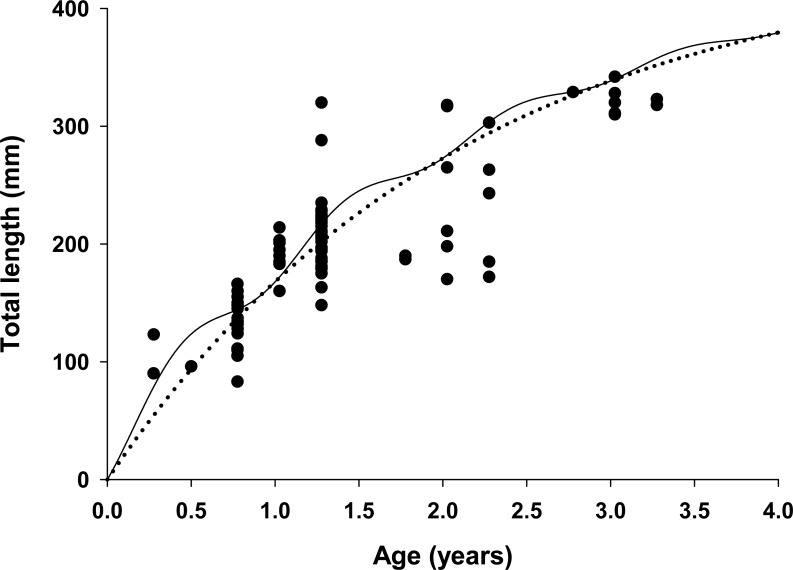
Estimated growth of lionfish using two growth models. Traditional (dotted line) and seasonalized (solid line) von Bertalanffy growth functions predicting size-at-age generated from the best fit traditional (Model 3) and seasonal (Model 1) models (see Table 1). The estimated ages of lionfish from sagittal otolith analysis are plotted as solid circles.

The best fit model allowed for seasonal growth (Tables 1 and 2; Figs. 2 and 4); traditional VBGF formulations generated poor fits to the data (Table 1). The estimated seasonalized VBGF from the model was:

(8) \begin{eqnarray*}{L}_{t}=448\mathrm{ mm} \left[ 1-{e}^{-0.47 \left( t-0 \right) + \left[ \frac{ \left( 0.61\ast 0.47 \right) }{2\pi } \sin \nolimits \,\pi \left( t-0.71 \right) \right] - \left[ \frac{0.61\ast 0.47}{2\pi } \sin \nolimits \,\pi \left( 0.71 \right) \right] } \right] .\end{eqnarray*}

All growth parameters were freely estimated with the exception of *L*_∞_ which was fixed at the maximum observed size of 448 mm TL, a common convention (Sammons & Maceina, 2009). Constraining *L*_∞_ was required because relatively few old fish were captured and the length data contained little information about maximum size. Consequently, the model produced biologically unrealistic estimates of *L*_∞_ in excess of 800 mm TL when this parameter was not constrained. This is a commonly reported problem since *K* and *L*_∞_ are negatively correlated and adequate fits can be achieved over a broad range of parameter values (Shepherd, 1987). We also effectively fixed *t*_0_ at 0 by removing it from the model; this nuisance parameter was not required since the time of birth (*t*_*b*_) was independently estimated within the model (Gulland & Rosenberg, 1992; Taylor, Walters & Martell, 2005). The estimated value for *C* was 0.61 indicating relatively strong seasonality in growth with slowest growth occurring on February 13th (*t*_*w*_ + *t*_*b*_ = 0.12). The seasonal VBGF for lionfish predicted patterns in external (not used in model fitting) observed length-frequency data collected in 2013 and 2015 well, although some evidence for interannual variation in growth is apparent (Fig. 3) and were in close agreement with size-at-age determined from otolith analysis (Fig. 4).

### Recruitment

Annual lionfish recruitment was estimated to occur as a single event in early summer (*t*_*b*_ = 0.41 = June 2nd). The distinct bimodal distribution of total lengths among early ages in all years is consistent with a brief period of annual recruitment (Figs. 2 and 3) and also with results from otolith aging where 94% and 75% of otoliths from lionfish collected in April and August, respectively, had the same marginal increment. Moreover, the observed marginal increment relative to annuli deposition was consistent with a birth date in summer.

### Population age structure

The lionfish population in northeast Florida was relatively young. Aged otoliths ranged from age 0 to age 3 (Fig. 4) with age 0, 1, 2, 3 fish comprising 9%, 69%, 14% and 9% of the population, respectively (Table 3). Otolith analysis identified no fish greater than 3 years of age supporting the model assumption of only four age classes in the population (Figs. 2 and 4). Older lionfish are probably more common than suggested by our data since the primary goal of otolith analysis was model validation and the largest fish aged in this study was smaller (342 mm TL) than the maximum size captured (448 mm TL). However, this bias is likely small since very few lionfish were larger than 342 mm TL (6.1%) and only 0.8% were greater than 400 mm TL. Model estimated population age structure generally agreed with the results from otolith aging (Table 3, Fig. 4), but direct quantitative comparisons between otolith samples and model outputs are not possible because we selectively aged larger fish, for which age is more uncertain, for model validation. For all months, the highest proportions of fish were age 1 and age 2 (Table 3). The highest proportion of age 0 fish occurred in April just prior to the cohorts estimated first birthday (*A*_*a*_ = 0.91) on June 2nd (*t*_*b*_ = 0.41; see above) when these fish, although not fully recruited to the fishery, were large enough (TL = 151 mm) to be effectively targeted by divers (Barbour et al., 2011; Edwards, Frazer & Jacoby, 2014). Newly recruited age 0 fish were largely absent during July and August, likely because fish at this age are translucent and cryptic and too small (TL = 32–59 mm) to be captured by spearfishers. Model predicted proportions at age indicate that older fish were rare in all months with Age 3 fish comprising only 6% of the population on average (Table 3).

**Table 3:** Age structure of the lionfish population from northeast Florida. The estimated proportion of the population in each age class (*P*_age_) in each sampling month from April 2014 to January 2015 estimated from the best fit model (Table 1). Mean proportions ($\overline{x}$) from monthly model estimates were calculated as a weighted average (∑(*P*_*a*,*m*_∗*n*)∕*N*, where *N* is the total number of fish captured in all months (*N* = 2, 137).

	*P*_*a*_				
Model	0	1	2	3	*n*
April	0.37	0.62	0.00	0.02	850
July	0.00	0.63	0.37	0.00	33
August	0.01	0.50	0.40	0.10	1,102
October	0.20	0.67	0.10	0.02	53
November	0.09	0.49	0.37	0.04	41
January	0.00	1.00	0.00	0.00	58
$\overline{x}$	0.16	0.56	0.22	0.06	
Otoliths	0.09	0.69	0.14	0.09	

## Discussion

This study developed and validated a length-based, age-structured model to estimate age, growth and population structure of lionfish from length-frequency data in northeast Florida. The results of the model provide some key insights: (1) lionfish in this region grow rapidly, (2) lionfish exhibit seasonal growth, (3) peak recruitment of lionfish to northeast Florida occurs during a short window in early summer, (4) the population of lionfish in northeast Florida is young with older fish virtually absent, likely reflecting the removal of older lionfish by spearfishers in combination with the movement of lionfish to deeper water with age. These findings add to our growing understanding of lionfish biology and provide the first estimates of lionfish vital rates in the region.

Lionfish grew exceptionally fast, much faster than in their native range (Pusack et al., 2016), mirroring the findings of many other studies (Potts, Berrane & Morris Jr, 2010; Barbour et al., 2011; Jud & Layman, 2012; Albins, 2013; Benkwitt, 2013; Akins, Morris Jr & Green, 2014; Edwards, Frazer & Jacoby, 2014; Fogg et al., 2015; Rodríguez-Cortés, Aguilar-Perera & Bonilla-Gómez, 2015; Pusack et al., 2016). Rapid growth is a trait positively correlated with invasibility and coupled with other life history information (Morris Jr, 2009; Ahrenholz & Morris, 2010; Côté et al., 2013) may help to explain the successful establishment and rapid invasion and of the species and is concerning for potential lionfish impacts in this region through both competition and predation. Other fish in the region that are competing on the same trophic level as lionfish (Layman & Allgeier, 2012) such as black sea bass (*Centropristis striatus*) and vermillion snapper (*Rhomboplites aurorubens*), take a longer time to grow to reproductive size (Hood, Godcharles & Barco, 1994; Zhao, McGovern & Harris, 1997). With both a low size at maturity and fast growth rates, lionfish have the potential to reach a large size and reproduce well before their native competitors.

**Table 4:** Summary of available growth estimates for lionfish in the invaded range. Von Bertalanffy growth parameters (*L*_∞_, *k*, *t*_0_), estimated size at age at 1, 2 and 3 years (${\overline{L}}_{1},{\overline{L}}_{2},{\overline{L}}_{3}$), observed maximum size (*L*_max_) and age (*L*_*a*_), and method of estimation for available studies of lionfish growth in the invaded and native range.

Location	Sex	*L*_∞_	*K*	*t*_0_	${\overline{L}}_{1}$	${\overline{L}}_{2}$	${\overline{L}}_{3}$	*L*_m*ax*_	*A*_max_	Method	Temp (°C)^a^	**Reference**
**Atlantic Ocean**												
North Carolina	Combined	455	0.32	−1.22	231	293	337	464	8.0	Otoliths	22.1	Potts, Berrane & Morris Jr (2010)
North Carolina	Combined	425	0.47	−0.50^b^	169	265	325	464	8.0	Otoliths	22.1	Barbour et al. (2011)^b^
Northeast Florida	Combined	448	0.47	0.00^b^	168	273	339	448	3.3^c^	Length-based	21.4	Present study
**Gulf of Mexico**												
Northeast	Combined^d^	393	0.54	−0.08	174	265	319	434^e^	4.5	Otoliths	21.8	Fogg et al. (2015)
West	Combined^d^	389	0.54	−0.34	200	279	325	434^e^	4.0	Otoliths	21.7	Fogg et al. (2015)
Southeast	Combined^d^	429	0.57	−0.16	208	304	358	434^e^	4.5	Otoliths	25.1	Fogg et al. (2015)
Yucatan	Combined	420	0.88	0.11	228	340	387	389	n/a	Length-based	29.3	Rodríguez-Cortés, Aguilar-Perera & Bonilla-Gómez (2015)
**Florida Keys**												
Key Largo	Combined	411	0.70	0.00^b^	207	310	361	452	n/a	Length-based	26.3	Swenarton, Johnson & Akins (2014)
**Caribbean Sea**												
Little Cayman	Female	286	0.57	−1.01^b^	195	235	257	333	3.0	Otoliths	30.0	Edwards, Frazer & Jacoby (2014)
Little Cayman	Male	382	0.38	−1.01^b^	204	260	299	391	5.0	Otoliths	30.0	Edwards, Frazer & Jacoby (2014)
Little Cayman	Combined^f^	349	0.42	−1.01^b^	199	250	284	391	5.0	Otoliths	30.0	Edwards, Frazer & Jacoby (2014)
Caymans/ Bahamas	Combined	322	1.48	−0.07^b^	256	307	319	n/a	n/a	Tagging	29.7/28.7	Pusack et al. (2016)
**Indo-Pacific**												
Philippines/ Marianas	Combined	225	1.62	−0.07^b^	175	215	223	n/a	n/a	Tagging	30.2/28.1	Pusack et al. (2016)
	$\overline{x}\hspace*{1em}$^g^	404.1	0.64	−0.33	204.0	288.6	335.4	429.7	4.9			
	*σ*	42.2	0.33	0.45	28.9	27.2	28.4	32.2	1.6			
	*CV*	10.4	52.2	138.7	14.1	9.4	8.5	7.5	33.4			

**Notes.**

a Average temperatures represent regional long-term annual averages and do not reflect conditions at study locations at the time they were conducted.

b Parameter value was fixed during growth curve fitting.

c Re-analysis of data obtained from Potts, Berrane & Morris Jr (2010).

d Maximum age adjusted for interval between estimated birth date and first annulus.

e *L*_max_ was not reported on a site-specific basis in Fogg et al. (2015) and represents combined data for all regions.

f The estimates reflect combined data from both sexes from Edwards, Frazer & Jacoby (2014).

g Values include combined data from the invaded range and only independent estimates (e.g., *A*_max_ from Potts, Berrane & Morris Jr, 2010 and Barbour et al., 2011 is only included once because they use the same data set).

Our reported values for *L*_∞_ and *K* are broadly consistent with previous studies (Table 4), although some variability exists across locations which may reflect differences in collection methods and sampling intensity, elapsed time since colonization, or regional differences in environmental or ecological factors. However, comparisons of individual growth parameters in isolation across regions can be problematic because *L*_∞_, *K*, and *t*_0_ are typically strongly correlated (Shepherd, 1987) and actual realized growth rates (size-at-age) are a combination of all three values (Gwinn, Allen & Rogers, 2010; Pardo, Cooper & Dulvy, 2013). To compare growth across regions directly, we used reported VBGF parameters from previous studies to calculate mean size-at-age for lionfish at age 1, 2 and 3 (${\overline{L}}_{1},\hspace*{1em}{\overline{L}}_{2},\hspace*{1em}{\overline{L}}_{3}$; Table 4). In particular, while *K* was variable across studies ($\overline{x}=0.64$; Coefficient of Variation (CV) = sd/$\overline{x}=52$%); these differences were reduced when comparing mean size-at-age from the VBGF (CV = 14, 9, and 8% for ${\overline{L}}_{1},\hspace*{1em}{\overline{L}}_{2},\hspace*{1em}{\overline{L}}_{3}$, respectively across all studies; Table 4). Two exceptions were studies in the southern Gulf of Mexico (Rodríguez-Cortés, Aguilar-Perera & Bonilla-Gómez, 2015) where lionfish grew faster than other locations and in the Cayman Islands (Edwards, Frazer & Jacoby, 2014) where growth was much slower. Rapid growth in the first study (Rodríguez-Cortés, Aguilar-Perera & Bonilla-Gómez, 2015) may be driven by the warmer temperatures in the southern Gulf of Mexico compared to where our study was conducted in northeast Florida (Table 4). Growth rates from northeast Florida were most similar to those in North Carolina (Potts, Berrane & Morris Jr, 2010; Barbour et al., 2011) and the northern Gulf of Mexico (Fogg et al., 2015) providing additional support for temperature as an important factor underlying variation in growth in this species (Table 4). Such a relationship would not be unusual; temperature is well known to strongly influence growth rates in fishes (Pauly, 1980; Gislason et al., 2010) and the seasonal growth observed in this study clearly demonstrates the effect of temperature on growth for lionfish. Conversely, the slower growth of lionfish in one study in the Cayman Islands (Edwards, Frazer & Jacoby, 2014) cannot be explained by temperature. Slow growth in this study may be explained by other environmental or ecological factors (although Pusack et al. (2016) demonstrate faster growth in this region), be an artifact of curve fitting procedures or result from the recency of colonization as discussed by the authors (Edwards, Frazer & Jacoby, 2014). Further studies of lionfish growth in the wider Caribbean will help to more clearly identify the various mechanisms underlying regional differences in growth rates.

We constrained *L*_∞_ to 448 mm TL, the largest fish observed in our data set (*n* = 2, 137) because unconstrained fits produced biologically unrealistic values (*L*_∞_ > 800 mm TL). In practice, this approach is often required when data contain few old fish and consequently little information about *L*_∞_ (Shepherd, 1987). In such cases, fixing *L*_∞_ to the maximum observed size (*L*_max_) is a common convention (Sammons & Maceina, 2009). Our value of *L*_8_ is similar to those predicted from empirically derived relationships between *L*_∞_ and size at maturity (190 mm TL; Gardner et al., 2015) and *L*_max_ (448 mm TL; this study) which are 421 and 450 mm TL, respectively (Froese & Biholan, 2000) and is also consistent with previously reported estimates of *L*_∞_ (Table 4) and with maximum reported lengths in the catch from the invaded range (476 mm TL; Morris Jr, 2012).

One key finding from our model was the strong support for seasonal growth rates (*C* = 0.61) which were correlated with seasonal fluctuations in bottom water temperatures (Table 1, Fig. 4). Temperature has a large effect on growth in fishes, resulting in seasonal cycles in growth rates in temperate areas and more uniform growth in tropical regions (Pauly, 1980). Our model estimated the winter point to be 0.12 which corresponds to a calendar date of February 13th. This is in strong agreement with known climatology for waters of the southeastern continental shelf of the US where bottom temperatures are typically coldest in late winter (Atkinson et al., 1983). Seasonal growth resulted in substantial changes in intra-annual size-at-age for young fish relative to the traditional VBGF; but not in overall annual estimates of growth (*K* = 0.47 for both seasonal and traditional VBGFs; Table 2, Fig. 4). The greatest difference was observed for age 0 fish which were estimated to be 38% larger than predicted by the traditional VBGF by late summer in their first year (111 mm vs 80 mm TL; Fig. 4). Seasonal growth could have important implications for predator–prey dynamics between lionfish and native fishes. Predation risk in marine fishes is typically inversely related to body size (Rice et al., 1993; Lorenzen, 1996) and this relationship can be particularly strong for the prey of gape-limited predators. However, seasonal growth is probably not likely to substantially impact predation on lionfish. While many of the native predators documented to consume lionfish are gape-limited, suction feeders (Nassau grouper, *Epinephelus striatus* (Maljković, Van Leeuwen & Cove, 2008; Diller, Frazer & Jacoby, 2014); tiger grouper, *Mycteroperca tigris* (Maljković, Van Leeuwen & Cove, 2008), and nurse sharks (*Ginglyostoma cirratum*, Diller, Frazer & Jacoby, 2014); direct observations of predation on lionfish are scarce and most evidence indicates that native predators are not effective at controlling lionfish populations (Hackerott et al., 2013; Valdivia et al., 2014). Conversely, seasonal growth in temperate regions, may allow gape-limited lionfish (Muñoz, Currin & Whitfield, 2011; Green et al., 2012) access to larger prey earlier in their life history than previously thought. While this finding does not significantly alter our understanding of lionfish trophic dynamics **because this effect is inherent in field observations of prey consumption, abundance and size-structure (Albins & Hixon, 2008; Green et al., 2012); seasonal growth should be considered in predictive models which use the VBGF to ensure accurate estimates of lionfish growth in temperate regions. For example, the predictions from bioenergetic (Cerino et al., 2013), removal efficacy (Barbour et al., 2011) and prey consumption models (Green et al., 2014) all rely on biological and ecological relationships that scale with lionfish body size (e.g., maximum prey size) or mass (e.g., consumption rates, metabolism) which are underestimated when assuming non-seasonal growth.

Although not important for tropical areas of the invaded range, seasonal growth may lower the risk of winter mortality for juvenile lionfish in temperate regions where winter water temperatures approach thermal minima for lionfish. Overwinter mortality in fishes can be substantial in coastal systems and is often size-selective with larger individuals more resistant to mortality from both thermal stress (Lankford & Targett, 2001) and starvation (Henderson, Holmes & Bamber, 1988; Hurst, 2007). Lionfish are a tropical species and generally intolerant of cold temperatures (Kimball et al., 2004). Thus, larger size at the onset of winter may at least partially mitigate the negative effects of low water temperatures in temperate regions.

Lastly, as fisheries for lionfish develop and intensify in the invaded range, resource managers may begin to apply various population and stock assessment models (i.e., catch curves, yield-per-recruit) to this species. Seasonal growth must be considered in many of these models, which generate biased outputs (e.g., natural mortality) and associated management reference points when assuming a traditional VBGF (Sparre, 1990; Pauly, 1990; Hufnagl, Huebert & Temming, 2013).

Distinct annual cohorts are clearly identifiable in our length-frequency data, particularly in months with large sample sizes (Figs. 2A, 2C and 3). Several non-mutually exclusive hypotheses could explain this observed pattern. One explanation is that peak spawning of lionfish in regions that contribute to recruitment in northeast Florida varies seasonally. Further, to coincide with the predicted timing of lionfish recruitment in our region, peak spawning in source populations would be need to occur in summer. Although lionfish are capable of spawning throughout the year (Morris Jr, 2009), most studies do provide support for increased reproductive activity in summer. Gardner et al. (2015) reported two peak spawning periods for lionfish in the Cayman Islands occurring in spring and late summer, and spawning activity also peaks in summer in the eastern Gulf of Mexico (Fogg, Brown-Peterson & Peterson, 2013), North Carolina and The Bahamas (Morris Jr, 2009). Source populations for larvae settling in northeast Florida are not definitively known, but evidence from population genetics (Freshwater et al., 2009; Butterfield et al., 2015), biophysical modeling (Cowen, Paris & Srinivasan, 2006; Johnston & Purkis, 2011; Johnston & Purkis, 2015) and chronology of invasion history (Schofield, 2009) suggest a combination of self-recruitment and subsidies from upstream locations such as southern Florida and northern Cuba. Unfortunately, little is known about reproductive biology of lionfish in these regions. Alternatively, recruitment of lionfish in summer may be enhanced by seasonal physical oceanographic conditions promoting the delivery and/or retention of larvae to the region or by seasonal differences in post-settlement survival, or a combination of these processes. Although the causative factors driving this recruitment cannot be determined from this study, identifying factors that may limit lionfish larval supply or survival is inherently important to lionfish management and control efforts (Johnston & Purkis, 2011; Johnston & Purkis, 2015; Morris Jr, Shertzer & Rice, 2011).

Both the observed otolith ages and model predicted age structure indicate that a majority of the sampled population in northeast Florida is three years of age or younger, a recurring pattern in numerous studies with lionfish (Barbour et al., 2011; Edwards, Frazer & Jacoby, 2014; Fogg et al., 2015). Lionfish live for decades in aquaria (Potts, Berrane & Morris Jr, 2010) and older fish are reported from North Carolina (Barbour et al., 2011) and the more recently invaded Caribbean (Edwards, Frazer & Jacoby, 2014), so this finding is unexpected given that lionfish have been established in northeast Florida for almost two decades (Schofield, 2009). The absence of older cohorts is unlikely to be explained by high natural mortality given their large size and a lack of evidence for substantial predation on lionfish by native fishes (Valdivia et al., 2014). The observed age-structure in our study is likely explained by a combination of non-mutually exclusive factors: (1) local culling activities that target larger lionfish; (2) ontogenetic movement of lionfish to deeper water over time; and (3) limitations our otolith analysis which was primarily designed to validate the growth model (although this effect is likely small relative to the others). Repeated culling may reduce the number of older, larger fish over time as is commonly observed in fishery species (Worm et al., 2009); shifts towards smaller size of lionfish at fished relative to unfished locations have been reported in several studies (De León et al., 2011; Frazer et al., 2012). Although lionfish removal efforts in northeast Florida have intensified in recent years, lionfish are not easily accessible (>15 km offshore) and overall directed effort for this species in this region is still low. Thus, regional fishing pressure certainly contributes to the truncated population age-structure but is not likely to be the sole driver. The absence of older lionfish in our study may also be partially explained by ecological factors. For example, the observed population age structure is consistent with an ontogenetic shift of older lionfish to deeper waters likely occurring in winter (older fish (2+) are common during summer and fall but almost completely absent in collections from January and April; Table 3). Ontogenetic shifts are well documented for many marine fishes (Eggleston, Dahlgren & Johnson, 2004; Eggleston, Grover & Lipcius, 1998; Johnson, 2004), and have been hypothesized to explain the presence of larger lionfish at depth (Barbour et al., 2011; Claydon, Calosso & Traiger, 2012; Swenarton, 2016). Surveys employing other gear types (e.g., ROVs, otter trawls) report that lionfish are abundant at depth in both the Gulf of Mexico (Nuttall et al., 2014; Switzer, Tremain & Keenan, 2015, Aguilar-Perera, Quijano-Puerto & Hernández-Landa, 2016) and western Atlantic Ocean (Meister et al., 2005), although none of the studies report lionfish size distributions. The presence of deep water refuges is a major concern for lionfish management and control, since lionfish residing at depth are inaccessible to spearfishers (currently the primary method of removal), and culling efforts in shallow depths may be replenished by larval export from lionfish at depth (Morris Jr, 2009; Green et al., 2014). If lionfish are moving to deeper waters as they grow, a major unresolved question is determining the proportion of deep water lionfish that are immigrants from shallow water relative to the proportion that initially settled at depth. Thus, further studies using traditional tagging, acoustic telemetry or biogeochemical approaches will be needed to address this question and should be prioritized.

Overall, our model generated biologically realistic parameters, provided good fits to observed length-frequency data, and was in close agreement with the results from otolith aging analysis (Fig. 4) and when fit to length-frequency data external to model (Fig. 3). The length-based approach described here may offer a more practical alternative to aging by otoliths alone which is time and labor intensive and can be particularly difficult for fishes in tropical regions which often lack defined annuli (Edwards, Frazer & Jacoby, 2014). Moreover, this approach requires only length data which are easily collected and widely available. Thus this approach could be applied to existing data from lionfish populations throughout the invaded range. Such spatially-explicit information on lionfish biology would aid management agencies seeking to develop effective localized removal and harvest strategies.

##  Supplemental Information

10.7717/peerj.2730/supp-1 Supplemental Information 1 Lionfish length-frequency data

## Additional Information and Declarations

### Data Deposition

The following information was supplied regarding data availability: The raw data has been supplied as a Supplemental Information 1.
